# The long-term results of temporary urethral stent placement for the treatment of recurrent bulbar urethral stricture disease

**DOI:** 10.1590/S1677-5538.IBJU.2015.0115

**Published:** 2016

**Authors:** Gokhan Temeltas, Oktay Ucer, Mehmet Bilgehan Yuksel, Bilal Gumus, Volkan Tatli, Talha Muezzinoglu

**Affiliations:** 1 Department of Urology, Faculty of Medicine - Celal Bayar University, Manisa, Turkey

**Keywords:** Urethral Stricture, Stents, Disease, Therapeutics

## Abstract

**Aim:**

To evaluate the long term outcomes of temporary urethral stent placement for the treatment of recurrent bulbar urethral stricture.

**Materials and Methods:**

Twenty-eight patients who underwent temporary polymer coated urethral stent placement due to recurrent bulbar urethral stricture between 2010 and 2014 were enrolled in the study. The long term outcomes of the patients were analyzed.

**Results:**

The mean age of the patients was 62.3±6.4 (44–81). The overall clinical success was achieved in 18 (64.2%) of the 28 patients at a median (range) follow-up of 29 (7–46) months. No patient reported discomfort at the stent site. Stone formation was observed at the urethral stent implantation area only in one patient. Stenosis occurred in the distal end of the stents in two patients and took place in bulbar urethra in seven patients after removed the stents. The mean maximum urine flow rates were 6.24±2.81mL/sec and 19.12±4.31mL/sec before and at 3 months after the procedure, respectively.

**Conclusion:**

In this study, the success rate of temporary urethral stent placement has remained at 64.2% at a median follow-up of 29 months. Therefore, our outcomes have not achieved desired success rate for the standard treatment of recurrent bulbar urethral stricture.

## INTRODUCTION

Urethral strictures are commonly managed initially by direct visual internal urethrotomy or dilatation. This can be curative in half of cases in a suitably located short stricture (1). Nonetheless, these approaches are associated with high recurrence rates and deficient long-term efficacy, especially for strictures longer than 1cm (2). Therefore, many patients progress to open reconstruction such as excision and primary anastomosis, penile skin graft or buccal mucosa graft urethroplasty, which provide better long-term outcomes (3). Although direct visual internal urethrotomy or dilatation is appealing both for urologists and patients as it is minimally invasive, urethroplasty is not a minimal invasive approach. Therefore, as an alternative treatment to traditional methods urethral stents have been used since 1985 in the treatment of urethral strictures and successful results have been reported by many centers (4). Temporary urethral stent placement for recurrent bulbar urethral strictures after direct visual internal urethrotomy or dilatation may be considered as an option before deciding urethroplasty.

The purpose of our study was to evaluate the long term outcomes of temporary urethral stent placement for the treatment of recurrent bulbar urethral stricture.

## MATERIALS AND METHODS

### Participants

Twenty-eight patients who were treated with temporary polymer coated urethral stent (Allium^®^, Allium LTD, Caesarea, Israel) due to recurrent bulbar urethral stricture between 2010 and 2014 were included in the study. All patients had previously undergone many dilatations and/or direct visual internal urethrotomies. The inclusion criteria included: adult patients, recurrent bulbar urethral strictures and at least two previous dilatations or direct visual internal urethrotomies. The exclusion criteria consisted of: penile or posterior urethral stricture, history of pelvic malignancy or radiation, and previous hypospadias repair.

The patients were evaluated with retrograde urethrogram and uroflowmetry. Residual urine volume was estimated with ultrasonography. Pre-operative demographics and clinical characteristics were recorded including age, stricture etiology, location, length, maximum urinary flow rate (Q_max_), number of previous direct visual internal urethrotomies/dilatations and time to last stricture recurrence. All patients provided informed consent form and underwent urethral stent placement. The study protocol was approved by the Local Ethics Committee.

## Surgical technique

All patients used 2^nd^ generation cephalosporins for prophylaxis. After induction of adequate spinal or local anesthesia, the patient was placed in the lithotomy position. Direct visual internal urethrotomy was performed at 12 o'clock direction and then the urethral stent was placed in bulbar urethra with 0 degree optical image. No Foley catheter was inserted after the urethral stent placement.

All patients were evaluated with uroflowmetry and post voiding residual urine measurement in the postoperative third month. All stents were removed at 3 or 6 months after the procedure. All patients were followed at 3, 6 and 12 months after stent removal and then yearly. The success criteria after stent removal were no evidence of stricture on urethrogram or endoscopy, urinary peak flow greater than 15mL/sec. and no recurrent urinary tract infection.

**Figure 1:**
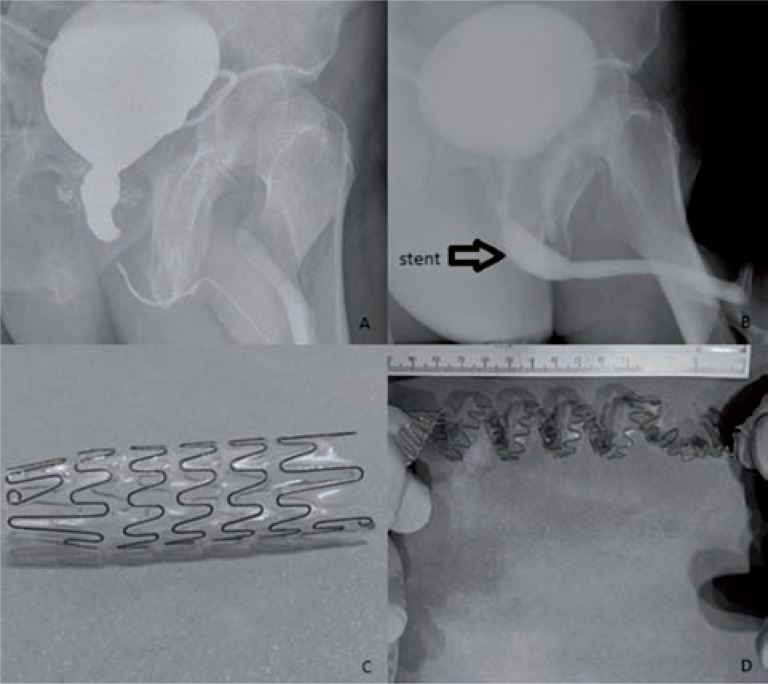
Radiologic images before and after the urethral stent replacement and, the views of stent before the replacement and after removal of it. **A)** The patient's voiding cystourethrography before the operation; **B)** The patient's retrograde urethrography after direct visual urethrotomy and replacement of stent; **C)** The view of stent before the replacement; **D)** The view of stent after removal of it.

A patient's radiologic image before and after the urethral stent replacement and the views of the stent before the replacement and after removal of it are given in Figure-1.

## Statistical analysis

Statistical analysis was performed using SPSS version 13.0. The paired Student's t-test was used to analyze and compare Q_max_ and post voiding residual urine preoperatively and at 3, 6 and 12 months after stent removal. P<0.05 was considered to indicate statistical significance.

## RESULTS

The mean age of the patients was 62.3±6.4 (44–81). The mean (range) stricture length was 1.9 (0.5–3.5)cm. The median (range) number of previous failed direct visual internal urethrotomy or dilatation was 3 (2–11). The mean (range) time to stricture recurrence was 5 (1–60) months after the most recent procedure. The etiologies of the strictures are summarized in Table-1.

**Table 1:** The etiologies of the strictures.

Etiology	Number of patients (%)
Iatrogenic	10 (36)
Trauma	7 (25)
Idiopathic	7 (25)
Post-infectious	4 (14)

All stents were inserted successfully. The operative time ranged between 15 to 35 minutes (25±5.15) with no obvious intraoperative complications. Spontaneous voiding was achieved in all patients immediately after stent insertion. No patient reported discomfort at the stent site. Stone formation was observed with infection at the urethral stent implantation area only in one patient two months after the operation. The stent was removed and given medication for infection. Stenosis occurred in the distal end of the stents in two patients and took place in bulbar urethra in seven patients after removal of the stents. The mean time from stent removal to restenosis was 4.2±1.5 (2–9) months. The overall clinical success was achieved in 18 (64.2%) of the 28 patients at a median (range) follow-up of 29 [7-46] months. Three stents were removed at 3 months after the operation because of stent migration. Urethral stenosis recurred in only one of these three patients. Other stents were removed at 6 months after the procedure.

The mean maximum urine flow rates were 6.24±2.81mL/sec and 19.12±4.31mL/sec before and at 3 months after the procedure, respectively. Preoperative and postoperative Q_max_ and post voiding residual urine values are given in Table-2.

**Table 2:** The mean Q_max_ and post voiding residual urine values in preoperative and postoperative follow-up.

	Pre-op (n=28)	Post-op	After stent removal				p value
		3 months (n=25)	3 months (n=23)	1 year (n=15)	2 years (n=9)	3 years (n=4)	
Q_max_ (mL/sec)	6.24*	19.12	19.01	18.40	18.56	17.26	<0.001
PVR (mL)	142.51*	40.12	44.14	46.85	41.56	48.89	<0.001

**PVR =** Post voiding residual urine;

* There were significantly differences between pre-op and post-op (both before and after stent removal)

**Q_max_ and PVR values**. There were no differences between post-op Q_max_ and PVR values.

## DISCUSSION

Urethral strictures may be cured with endoscopic technique alone. However, in longer strictures with significant spongiofibrosis, the natural history is of stricture recurrence. Heyns et al. (5) analyzed the role of repeated urethrotomies in patients who had a stricture recurrence after the first urethrotomy. They showed that after a single dilatation or a direct visual internal urethrotomy, not followed by restricturing at 3 months, the stricture recurrence rate was 55-60% at 24 months and 50-60% at 48 months. After a second direct visual internal urethrotomy for stricture recurrence at 3 months, the stricture-free rate was 30-50% at 24 months and 0-40% at 48 months. After a third dilatation or direct visual internal urethrotomy for stricture recurrence at 3 or 6 months, the stricture-free rate at 24 months was 0. Therefore, for the treatment of recurrent urethral strictures after many internal urethrotomies or dilatations it was recommended to perform urethroplasy. The success rate of urethroplasty with buccal mucosa was noticed to be 86% even if the length of stricture was long (mean 4.6cm) (6). In our country, costs of urethroplasty with buccal mucosa, direct visual urethrotomy alone and placement of urethral stent with urethrotomy are approximate 400 USD, 200 USD and 1500 USD (stent: 1300 USD, procedure: 200 USD), respectively. Urethroplasty and direct visual urethrotomy are more cost–effective than placement of stent in our country. However, disadvantages of these two approaches in recurrent urethral strictures, the success rate of direct visual urethrotomy is low and urethroplasty is not a minimal invasive approach.

Being an alternative approach, temporary urethral stent is suggested for these patients who have undergone many internal urethrotomies or dilatations (1, 7). Wong et al. (1) hypothesized that a temporary urethral stent might have a role to play in the management of recurrent urethral strictures if deployed early during endoscopic management. The temporary stent could act as a scaffold to splint against the mechanical forces of scar contraction during the healing phase. This may ultimately stabilize the stricture site during epithelization and thereby reduce the need for further endoscopic or urethroplasty procedures. Short-term stent placement also has the advantage of fewer complications, e.g. migration, discomfort, incontinence, infection and encrustation. Indeed, Atesci et al. (8) reported high complication rate of permanent Memotherm^®^ urethral stent in recurrent bulbar urethral strictures (discomfort in implantation area: 40%, partial stent migration: 10%, stone formation in implantation area: 10% and dripping after micturition: 75%). They also found that the overall success rate of permanent urethral stents was 87.5% at the end of the tenth year. Similar to our study, Sertcelik et al. (9) noticed long-term results of permanent urethral stent (Memotherm^®^) in the management of recurrent bulbar urethral stenosis. They observed that the success rate of stent was 78.7%, but also the complications rate after the operation was high (partial stent migration: 4.3%, hyperplastic reaction: 14.9%, discomfort in implantation area: 42.6%, post-micturition dribbling: 68.1%, pain during erection: 6.4%). These two studies show that the success rate of permanent stent is higher than direct visual internal urethrotomy in recurrent urethral strictures, but also the complication rate after placement of stent is very high. Stone formation of permanent stent has rarely been noticed in literature (10). Karakose et al. (10) reported the management of stone formation in the Memotherm^®^ urethral stent implantation area.

Yachia et al. (11) published their experience of using a UroCoil™ temporary stent in 172 patients with recurrent urethral strictures. The mean stent indwelling time was 12 months and their success rate was 83% at 24 months. In contrast to this study, Choi et al. (12) noticed that the success rate of covered nitinol stent in 33 patients was 55% and they found that leaving the stent for a minimum of 4 months resulted in less stricture recurrence. Wong et al. (1) used Memokath^®^ stent in patients (n=22) with recurrent bulbar urethral stricture and the success rate was found to be 78%. They removed the stents at three months after the operations and the mean follow-up period of their study was 23 months. Jordan et al. (13) placed a Memokath^®^ 044TW stent into bulbar urethra in patients (n=63) with recurrent urethral stricture and removed the stents at 12 months after the operation. They compared between stent and control groups. They reported that in stented patients patency was maintained significantly longer than controls (median 292 versus 84 days). However, they did not test stent durability and did not follow the patients after removal of the stents. The reason for conflicting results of above studies may be due to methodological diversity such as features of stents and removal time of stent.

The last study of the use of temporary urethral stent in literature was reported by Culha et al. (7). Similar to our study, they used Allium^®^ urethral stent. However, they removed the stents 3 or 18 months after stent insertion. Our time for stent removal was 3 or 6 months. Their success rate was reported 81.4% at a median follow-up of 10.6-month. In present study, the overall clinical success was found to be 64.2% at a median follow-up of 29 months. Although number of patients in our study was lower than their study, the mean follow-up period of our study was longer than their follow-up period. The low success rate of our study may due to that the mean time of stent removal in our study was lower than their study. They reported that longer indwelling time was statistically related to higher clinical success compared to shorter period. However, we think that prolonged stenting may increase the number of complications similar to the complication rates of permanent stents. In our study, the most serious complication was stone formation with infection on the stent and it was observed only in a patient. This patient had undergone direct visual internal urethrotomies 11 times. This complication may due to many previous urethrotomies. Further randomized studies should compare the complications and success rates between short–term and prolonged stenting.

## CONCLUSIONS

In our study, the success rate of temporary urethral stent placement has remained at 64.2% at a median follow-up of 29 months. Therefore, our outcomes have not achieved the desired success rate for the standard treatment of recurrent bulbar urethral stricture. The overall success rate of our study was lower than other studies. This may due to that the stent removal time of our study was lower than the others. We suggest that further studies should investigate the best time of stent removal in temporary stents for treatment of recurrent urethral stents. We could not compare between the success rate of temporary stent in our study and success rate of urethroplasty, because the number of patients in our study was too small for this comparison. Therefore, we also suggest that large population studies should compare between success rates of urethral stent and urethroplasty.

## References

[1] Wong E, Tse V, Wong J (2014). Durability of Memokath™ urethral stent for stabilisation of recurrent bulbar urethral strictures–medium-term results. BJU Int.

[2] Santucci R, Eisenberg L (2010). Urethrotomy has a much lower success rate than previously reported. J Urol.

[3] Bullock TL, Brandes SB (2007). Adult anterior urethral strictures: a national practice patterns survey of board certified urologists in the United States. J Urol.

[4] Braf Z, Chen J, Sofer M, Matzkin H (1996). Intraprostatic metal stents (Prostakath and Urospiral): more than 6 years' clinical experience with 110 patients. J Endourol.

[5] Heyns CF, Steenkamp JW, De Kock ML, Whitaker P (1998). Treatment of male urethral strictures: is repeated dilation or internal urethrotomy useful?. J Urol.

[6] Lumen N, Oosterlinck W, Hoebeke P (2012). Urethral reconstruction using buccal mucosa or penile skin grafts: systematic review and meta-analysis. Urol Int.

[7] Culha M, Ozkuvanci U, Ciftci S, Saribacak A, Ustuner M, Yavuz U (2014). Management of recurrent bulbar urethral stricture-a 54 patients study with Allium bulbar urethral stent (BUS). Int J Clin Exp Med.

[8] Atesci YZ, Karakose A, Aydogdu O (2014). Long-term results of permanent memotherm urethral stent in the treatment of recurrent bulbar urethral strictures. Int Braz J Urol.

[9] Sertcelik MN, Bozkurt IH, Yalcinkaya F, Zengin K (2011). Long-term results of permanent urethral stent Memotherm implantation in the management of recurrent bulbar urethral stenosis. BJU Int.

[10] Karakose A, Atesci YZ, Aydogdu O (2014). The stone formation in the Memotherm urethral stent implantation area: Is it a rare complication?. Can Urol Assoc J.

[11] Yachia D, Markovic Z, Markovic B, Stojanovic V (2007). Endourethral prostheses for urethral stricture. Acta Chir Iugosl.

[12] Choi EK, Song HY, Shin JH, Lim JO, Park H, Kim CS (2007). Management of recurrent urethral strictures with covered retrievable expandable nitinol stents: long-term results. AJR Am J Roentgenol.

[13] Jordan GH, Wessells H, Secrest C, Squadrito JF, McAninch JW, Levine L (2013). Effect of a temporary thermo-expandable stent on urethral patency after dilation or internal urethrotomy for recurrent bulbar urethral stricture: results from a 1-year randomized trial. J Urol.

